# CAR T Cells Beyond Cancer: Hope for Immunomodulatory Therapy of Infectious Diseases

**DOI:** 10.3389/fimmu.2019.02711

**Published:** 2019-11-21

**Authors:** Michelle Seif, Hermann Einsele, Jürgen Löffler

**Affiliations:** Department of Internal Medicine II, University Hospital Wuerzburg, Würzburg, Germany

**Keywords:** infectious diseases, mAb engineering, CAR T cells, HIV, HCV, CMV, invasive aspergillosis, HBV

## Abstract

Infectious diseases are still a significant cause of morbidity and mortality worldwide. Despite the progress in drug development, the occurrence of microbial resistance is still a significant concern. Alternative therapeutic strategies are required for non-responding or relapsing patients. Chimeric antigen receptor (CAR) T cells has revolutionized cancer immunotherapy, providing a potential therapeutic option for patients who are unresponsive to standard treatments. Recently two CAR T cell therapies, Yescarta® (Kite Pharma/Gilead) and Kymriah® (Novartis) were approved by the FDA for the treatments of certain types of non-Hodgkin lymphoma and B-cell precursor acute lymphoblastic leukemia, respectively. The success of adoptive CAR T cell therapy for cancer has inspired researchers to develop CARs for the treatment of infectious diseases. Here, we review the main achievements in CAR T cell therapy targeting viral infections, including Human Immunodeficiency Virus, Hepatitis C Virus, Hepatitis B Virus, Human Cytomegalovirus, and opportunistic fungal infections such as invasive aspergillosis.

## Introduction

Viral and opportunistic fungal infections represent a major threat to chronically infected individuals and immunocompromised patients. Despite the availability of antifungal and antiviral drugs, the mortality rate is still significant in high-risk patients (1–3). Current anti-viral treatments fail to cure chronic viral infections (caused by, e.g., HIV, HBV, and HCV) due to the viral-reservoir composed of infected cells that can stay latent for several years and would restart producing infectious virus at any time (4, 5) and the occurrence of resistance (6, 7). Therapies providing long term control or able to eradicate the viral-reservoir are required.

Pathogen-specific effector T cells play a crucial role in the control of acute viral and fungal infections in immunocompetent individuals (8–12), making adoptive T cell therapy an attractive alternative to currently used anti-infectious therapies. Pathogen-specific T cells occur in low frequencies in the patient's blood, making them difficult to isolate and expand. Moreover, they have exhausted phenotypes and might be rendered inefficient by viral escape mutation mechanisms lowering the major histocompatibility complex (MHC) or mutating the targeted epitope (10, 13–15). Thus, Chimeric antigen receptors (CARs) T cells present an attractive alternative.

CAR T cells are considered as a major scientific breakthrough and an important turning point in cancer immunotherapy (16), especially in the treatment of B cell malignancies. Recently, the US Food and Drug Administration (FDA) then the European Commission have approved two CAR T-cell products, Kymriah® (Novartis) and Yescarta® (Kite Pharma/ Gilead) for the treatment of B-cell precursor acute lymphoblastic leukemia and aggressive B-cell lymphoma, respectively. CAR T cells are described as having the targeting specificity of a monoclonal antibody combined with the effector functions of a cytotoxic T cell (17). They offer potential advantages over pathogen-specific T cells, the CAR allows antigen recognition independent of the MHC and can be designed to specifically target the conserved and essential epitopes of the antigen, which allows them to overcome pathogen escape mechanisms.

Few anti-infectious CARs were described in the literature so far, most of them targeting HIV. Here we review the progress and discuss the remaining challenges of making CAR T cell therapy a reality for individuals suffering from infectious diseases. The main anti-infectious CAR constructs are summarized in Table 1.

**Table 1:** CAR design of the most promising anti-infectious CAR T-cells.

**Pathogen**	**Targeted antigen**	**Targeting element**	**Spacer**	**Transmembrane domain**	**Costimulatory domain**	**Extra modification**	**References**
HIV	CD4 binding site on gp-120	CD4	n.a.	CD4	n.a.	C46 peptide	(18)
	CD4 binding site on gp-120	CD4	n.a.	CD4	n.a.	CCR5 sh 1005; sh 516	(19)
	CD4 binding site on gp-120	CD4	n.a.	CD8α	CD28 or 4-1BB		(20)
	CD4 binding site on gp-120	VRC01-scFv	(GGGGS)_3_	CD8α or CD28	CD28-4-1BB		(21)
	CD4 binding site on gp-120	105-scFv	CD8 hinge	CD3ζ	n.a.		(22)
	Env/gp120 glycans	CD4/ CRD	CD28	CD28	CD28		(23)
	V1/V2 glycan loop	PGT145-scFv	CD8α Hinge	CD8α	4-1BB	AAV6-CCR5	(24)
	CD4-induced epitope on gp120/CD4 binding site	17b-scFv/CD4	Tripeptide AAA	CD28	CD28		(25)
	CD4-induced epitope on gp120/CD4 binding site	mD1.22-G_4_S-m36.4	CD8	CD8	4-1BB	C46 peptide	(26)
HBV	S HBV surface protein	C8-scFv	IgG1 Fc	CD28	CD28		(27–29)
	HBV surface antigen	19.79.6-scFv	IgG4 Fc mutated	CD28	CD28		(30)
HCV	HCV E2 glycoprotein	e137-scFv	IgG Fc	CD28	CD28		(31)
CMV	Glycoprotein B	27-287-scFv	Ig Hinge	CD28	CD28		(32–34)
	Virally encoded FcRs	IgG1 or IgG4 Fc mutated	n.a.	CD28	CD28		(35)
*Aspergillus fumigatus*	β-glucan	Dectin 1	IgG4 Fc mutated	CD28	CD28		(36)

## CAR T Cells

CARs are synthetic receptors composed of a targeting element linked by a spacer to a transmembrane domain followed by an intracellular signaling domain. The targeting element is usually, but not exclusively composed by a single-chain variable fragment (scFv) (17). The spacer constitutes mainly of a full-length Fc receptor of an IgG (Hinge-CH_2_-CH_3_) or shorter parts like the Hinge region only or Hinge-CH_2_ (37–40). Furthermore, parts of the extracellular domains of CD28 and CD8α were used as spacers (41, 42). Several transmembrane domains were used to anchor the receptor on the surface of a T cell, mainly derived from CD28, CD8α, or CD4 (42–44). The signaling domain consists of the intracellular part of CD3ζ from the TCR complex (45). Over the years, in order to improve the CAR functionality and persistence, several generations of CARs have been established differing in their intracellular signaling (17). First-generation CARs mediated T-cell activation only through the CD3ζ complex (45, 46). Second-generation CARs include an intracellular costimulatory domain, mainly CD28 or 4-1BB, leading to an enhanced expansion, and functionality (43, 47–52). These second-generation receptors are the origin of the recently approved CAR T-cell therapies (53). Third-generation CARs combine two costimulatory domains, mainly CD28, and 4-1BB (54). Finally, fourth-generation CARs, also called TRUCKs (T-cells redirected for universal cytokine-mediated killing), emerged, including an additional transgene for inducible cytokine secretion upon CAR activation [mainly IL-12 (55)]. Several other strategies for minimizing toxicity and enhancing versatility and control of CAR T cells were reviewed by others (17, 56).

## CAR T Cells Specific for Human Immunodeficiency Virus (HIV)

Studies on developing CAR T cell therapy to cure HIV infections are ongoing since the early 90th. The first findings were already reviewed by others (57–60). Here we shortly summarize the anti-HIV CAR T cell history and focus on the most recent achievements.

### CD4 Based CARs

The concept of CAR T cells was initially described in the 90th when the cytotoxic T cells specificity was redirected toward HIV infected cells. The first CAR was specific for HIV envelope protein (Env) using the CD4 receptor as a targeting element fused to the CD3ζ chain for intracellular signaling (CD4ζCAR) (61, 62). Clinical trials with the CD4ζCAR showed that the concept is feasible and safe, but failed to reduce HIV viral burden permanently (63–66).

To improve the CAR T cell activity and persistence, CD4ζCAR was re-engineered into second-generation and third-generation CARs. While CAR T cells containing CD28 costimulatory domain promoted higher cytokine production and better control over HIV replication *in vitro*, the 4-1BB containing CARs were more potent in controlling HIV infection *in vivo*. When compared to first-generation CAR T cells, second-generation CAR T cells were more potent at suppressing HIV replication *in vitro*. Furthermore, in a humanized mouse model of HIV infection, they preserved the CD4^+^ T cell count, reduced HIV burden, and expanded to a greater extent than first-generation CAR T cells (20).

However, it was shown that CD4-based CARs render the CAR T cells susceptible to HIV infection (18, 25). To overcome this limitation, CD4ζCAR was equipped with either a viral fusion inhibitor (C46 peptide) (18) or small hairpin RNAs to knock down HIV-1 co-receptor CC-chemokine receptor 5 (CCR5) and degrade viral RNA (19). Both methods successfully rendered CD4ζCAR T resistant to HIV infection and conferred them a long persistence and proper control of HIV infection *in vivo* (18, 19).

Moreover, several genome editing techniques were used to knock out CCR5 in T cells to confer them permanent resistance to HIV infection (67). These include the use of ZFNs (Zinc-finger nucleases) (68), which showed promising results in clinical trials (NCT00842634, NCT01044654, NCT01252641), TALEN (Transcription activator-like nucleases) (69, 70), and CRISPR-CAS 9 (71) in preclinical studies. These endonucleases were already used to produce universal CAR T cells by knocking down the TCR (72–77). It would be useful to test them to knock down CCR5 in HIV-CAR T cells.

### scFvs Based CARs

To avoid using the CD4 as targeting element, novel CARs of several generations were designed using single-chain variable fragments (scFv) derived from broadly neutralizing antibodies (bNAbs) targeting Env.

Targets included the CD4-binding site, several antigens of glycoprotein 120 (gp120), the membrane-proximal region of gp41, the mannose-rich region, and variable glycan regions (20, 21, 24, 78).

Second-generation CARs for the different targets enabled the CAR T cells to kill HIV-1-infected cells. However, their antiviral activity was variable according to the virus strain (78). Second-generation anti-glycan CARs, in combination with CCR5 ablation, provided better control of viral replication than the CAR alone (24).

First-generation anti-gp120 CARs induced efficient activation and cytokine secretion by the gene-modified T cells and mediated lysis of envelope-expressing cells and HIV-1-infected CD4^+^ T-lymphocytes *in vitro* (22). Third generation anti-gp120 CAR-T cells were more efficient than CD4 based CARs in lysing gp120 expressing cells *in vitro*. Furthermore, their interaction with cell-free HIV did not result in their infection. More importantly, they efficiently induced cytolysis of the reactivated HIV reservoir isolated from infected individuals. Thus, anti-gp120 third-generation CAR T cells might be a suitable candidate for therapeutic approaches aiming to eradicate the HIV reservoir (21).

However, one major drawback to developing scFvs-based CAR T cell therapy is the HIV viral escape mutation mechanism that can abrogate the antibody-binding site and render the CAR T cell therapy inefficient.

### Bi- and Tri-specific CARs

In order to overcome the HIV mutation escape mechanism, bi-and tri-specific CAR-expressing T cells targeting up to three HIV antigens were designed to increase the specificity and affinity.

The CD4 segment was fused with an scFv specific for a CD4-induced epitope on gp120 (25) or the carbohydrate recognition domain (CRD) of a human C- type lectin binding to conserved glycans on Env (23). The CD4-anti gp120 scFv bispecific CAR had better suppressive activity against HIV than the CD4 alone. CD4-mannose binding lectin (MBL) CARs showed the best potency when compared to both CD4 alone and CD4-anti gp120 (23). However, since C- type lectins can bind glycans which are not specific for HIV infected cells and can be associated with healthy cells, off-targets cannot be excluded.

More recently, T cells were engineered with up to three functionally distinct HIV envelope-binding domains to form bispecific and tri-specific targeting anti-HIV CAR-T cells. These cells carry two distinct CARs expressed on one T cell or one CAR having tow targeting elements linked together. Targets included CD4-binding site on HIV gp120 and CD4-induced (CD4i) epitope on gp120 near the co-receptor binding site. Tri-specific CARs expressed the C46 peptide, which inhibits HIV viral fusion and thus can prevent the infection of CAR T cells. Bi-and tri-specific CAR T cells showed potent *in vitro* and *in vivo* anti-HIV effects, they efficiently killed HIV-infected cells in a humanized mouse model while protecting the CAR- T cells from infection (26).

Despite all the challenges faced, anti-HIV CAR T cell therapy made much progress toward enhancing the CAR T cell antiviral activity, protecting CAR T cells from HIV infection, and overcoming HIV escape mechanisms. Currently, at least two clinical trials are ongoing for latent reservoir eradication, one using a modified bNAb-based CAR-T cell therapy (NCT03240328) and one using CD4-based CAR-T cell therapy with CCR5 ablation (NCT03617198).

## CAR T Cells Specific for Hepatitis B Virus (HBV)

Some preclinical studies are focusing on engineering second-generation CAR T cells to cure chronic hepatitis B and prevent the development of hepatocellular carcinoma (HCC). Cytotoxic T cells were redirected toward HBV surface and secreted antigens.

Second generation CAR T cells were designed to target HBV-surface proteins S and L, which are expressed continuously on the surface of HBV replicating cells. S and L specific CAR T cells were able to recognize soluble HBsAg and HBsAg-positive hepatocytes *in vitro* and subsequently secret IFNγ and IL-2. S-CAR T cells were activated faster and secreted higher cytokine levels than L-CAR T cells. This might be due to the higher expression of the S-protein on the surface of viral and subviral particles when compared with the L-protein (27).

Furthermore, both CAR T cells were able to lyse HBV transfected cells as well as selectively eliminated HBV-infected primary hepatocytes. However, even after the elimination of HBV-infected hepatocytes, HBV core protein and HBV rcDNA remained detectable. It is most probably because HBV rcDNA is localized in viral capsids and thus protected from caspase-activated DNAses (27). The S-CAR construct was tested *in vivo* in an immune-competent HBV transgenic mouse model. CD8^+^ mouse T cells expressing the human S-CAR localized to the liver and effectively reduced HBV replication, causing only transient liver damage. Furthermore, contact of CAR T cells with circulating viral antigen did not lead to their functional exhaustion or excessive liver damage. However, the survival of the CAR T cells was limited due to the immune response triggered by the human CAR (28). In an immunocompetent mouse model tolerized with a signaling-deficient S-CAR, S-CAR T cells persisted and showed long-lasting antiviral effector function (29). However, the use of a transgene instead of cccDNA to transcribe HBV makes these mouse models unsuitable to judge whether S-CAR T cells can cure HBV infection (28, 29).

More recently, other novel second-generation CARs targeting HBsAg were designed with different spacer length. Only HBs -CAR T-cells equipped with a long spacer (HBs-G4m-CAR) recognized HBV-positive cell lines and HBsAg particles *in vitro* and subsequently produced significant amounts of IFN-γ, IL-2, and TNF-α. However, HBs-G4m-CAR T cells were not capable of killing HBV-positive cell lines *in vitro*. This might be due to HBsAg particles produced by HBV-positive cells that can bind to HBs-G4m-CAR T-cells and potentially inhibit CAR-T targeting or killing of infected cells. In a humanized HBV-infected mouse model, adoptive transfer of HBsAg-CAR T-cells led to the accumulation of the cells in the liver and an important reduction in plasma HBsAg and HBV-DNA levels. Furthermore, the absence of HBV core expression in a portion of human hepatocytes and the unchanged plasma human albumin levels indicated HBV clearance without destruction of the infected hepatocytes. However, no complete elimination of HBV was observed. Despite this limitation, HBs-G4m-CAR T cells had superior anti-HBV activity than HBV entry inhibitors (30).

These studies showed promising results; a direct comparison of S-CAR T cell and HBsAg-CAR T-cell would be interesting to test. Furthermore, a better mouse model more representative of the actual infection should be used to evaluate the CAR activity *in vivo*. Finally, combination therapy using CAR T-cells with reverse transcriptase inhibitors or hepatitis B immunoglobulin might be required to have better control of the HBV infection.

## CAR T Cells Specific for Hepatitis C Virus (HCV)

Very recently, the first two CARs targeting HCV were designed based on a broadly cross-reactive and cross-neutralizing human monoclonal antibody specific for a conserved epitope of the HCV E2 glycoprotein (HCV/E2). Anti-HCV CAR T cells showed good anti-viral activity and lyzed HCV/E2-transfected as well as HCV-infected target cells (31).

This study showed that the concept of CAR T cells might also be suitable for the treatment of HCV. The described CAR should be evaluated *in vivo* in a suitable animal model. Furthermore, since HCV/E2 is the main target of the host immune response and is consequently very susceptible to mutations (32), targeting other conserved, and essential antigens might also be of interest.

## CAR T Cells Specific for Human Cytomegalovirus (CMV)

The first CAR targeting CMV was described in 2010 based on the anti-gB antibody. Second generation gB CAR T cells were activated when co-cultured with CMV-infected cells and secreted TNF α and IFN γ and subsequently inhibited CMV replication in infected cells (33–35). Moreover, they eliminated gB transfected cells (33) but were not always able to lyse infected cells, especially at later stages of the replication cycle. This might be due to HCMV-encoded anti-apoptotic proteins that are known to prevent the suicide of infected host cells (34, 35). This CAR T cell therapy was not tested *in vivo* due to the few sequence similarities between the murine CMV gB protein and the human one. An appropriate mouse model using a recombinant MCMV expressing HCMV-gB should be developed (33).

In a later study, it was shown that the long spacer (CH2–CH3 Fc domain from IgG1) usually used in CAR preparation could bind to virally encoded Fc binding receptors on the surface of infected cells and act as a receptor for CMV. The mutated form of the spacer is only recognized by viral FcRs and not the human ones. In this way, the long spacer can act as a receptor for CMV infected cells (35).

The gB-CAR with long and short spacer should be further tested *in vivo* in an appropriate animal model. More targeting elements should be tested. Finally, the combination of new targeting elements with a long spacer might confer a bispecific targeting of CMV infected cells.

## CAR T Cells Specific for Epstein-Barr Virus (EBV)

To target Epstein-Barr virus (EBV) associated malignancies, a second-generation CAR specific for the EBV latent membrane protein 1 (LMP1) was described. EBV-CAR T cells were activated *in vitro* in co-culture with nasopharyngeal carcinoma cells overexpressing LMP1 and subsequently produced IFNɤ and IL-2. Intra-tumoral injection of EBV-CAR T cells in a xenograft mouse model having tumors overexpressing LMP1 reduced tumor growth (79).

CAR-T cell therapy for solid tumors is still facing many challenges, like the inability to reach the tumor and survive in the tumor microenvironment. These challenges and the developed strategies to overcome them were reviewed by others (80).

## CAR T Cells Specific for *Aspergillus fumigatus*

A second-generation CAR using the extracellular domain of Dectin-1 as targeting element called D-CAR was designed to target *Aspergillus fumigatus*. Dectin 1 is a C-type lectin receptor specific for ß-glucan, a motif expressed on the surface of many fungi (81). D-CAR T cells were activated by ß-glucan and subsequently secreted IFNγ and induced hyphal damage *in vitro*. In an immunocompromised invasive aspergillosis mouse model, D-CAR T cells reduced the fungal burden (36).

This study suggested that the application of CAR T cells might extend beyond cancer and chronic viral infections to acute fungal infections. Although promising results were shown for D-CAR T cells, Dectin 1 might not be the best targeting element to redirect the T cell specificity toward *Aspergillus fumigatus*. Since ß-glucans are not specific for *Aspergillus fumigatus* but rather a broad range of commensal and pathogenic microorganisms, off-target activity of the CAR T cells cannot be excluded (82). Using scFvs derived from fungal specific antibodies might provide better specificity and activity of the CAR. Moreover, strategies to significantly shorten the CAR T cell preparation time [currently time from leukapheresis to infusion of the CART product can take up to 3–4 weeks (83)] will be essential to allow their clinical use for acute infections.

## Conclusion and Perspectives

CAR T-cell therapy has gained much interest since its clinical application was approved for cancer immunotherapy. Relying on the knowledge accumulated on CAR T cell engineering in cancer research, many efforts are being made toward developing similar therapies for patients affected by chronical viral and acute invasive fungal infections. While targets are more precise and unique to the pathogen, making it easier to avoid off-targets, pathogen escape mechanisms, and reservoirs are still major obstacles.

**Figure 1:**
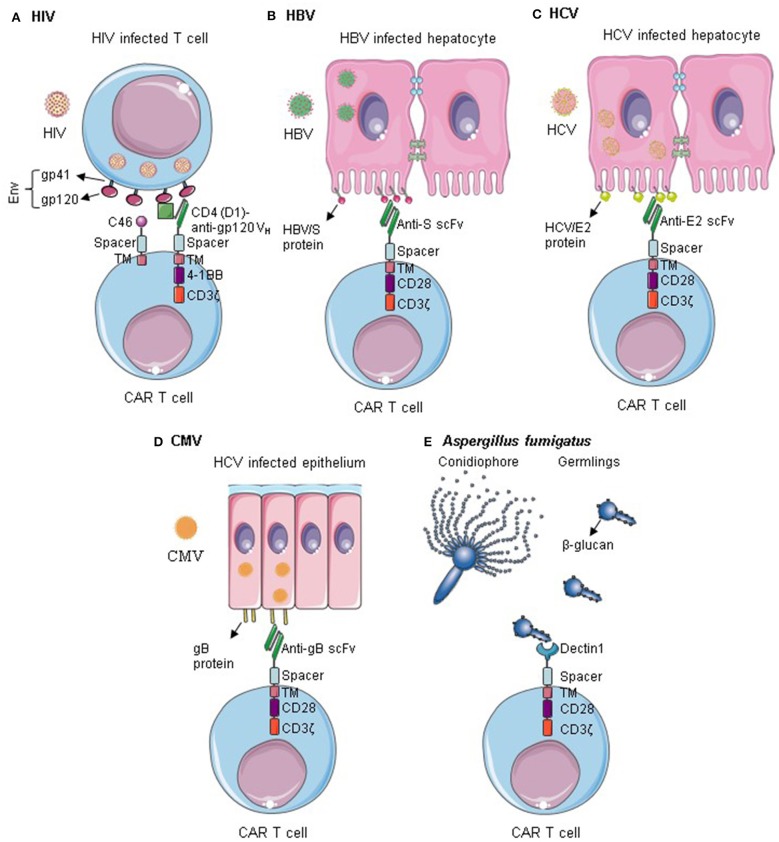
CAR T cells targeting infectious diseases. **(A)** T cells are redirected against HIV by the expression of Env-specific CARs on their surface. Additionally, they are rendered resistant to HIV infection by expression of an anti-fusion peptide. Anti-HIV CAR T cells can successfully kill HIV infected cells and control HIV infection. **(B–D)** CAR T cells specific for HBV S protein, HCV/E2, or gB can recognize cells infected by HBV, HCV, and CMV, respectively. They can selectively kill the infected cells within the epithelium. **(E)** Dectin 1-CAR T cells can directly bind to *Aspergillus fumigatus* germlings and induce hyphal damage. Env, HIV envelope protein; Gp, Glycoprotein; TM, transmembrane; V_H_, variable heavy chain; gB, Glycoprotein B. Some illustrations were obtained and modified from Servier Medical Art by Servier, licensed under Creative Commons Attribution 3.0 Unported License.

Several CARs targeting infectious diseases have been described; the most relevant ones are summarized in Figure 1 and Table 1. Tremendous progress was made in anti-HIV CAR T cell therapy, which reached now clinical trials. CAR T cells targeting other viruses such as HBV, HCV, CMV, and opportunistic fungus are still in their early pre-clinical testing. So far, promising data were observed, providing a proof of concept of CAR T cell application. Nevertheless, considerable optimization work is still required regarding the safety and efficacy of the constructs. More targets should be evaluated *in vitro* and *in vivo* in relevant animal models.

## Author Contributions

MS wrote the manuscript. JL and HE reviewed and edited the manuscript. All authors approved the manuscript for publication.

### Conflict of Interest

The authors declare that the research was conducted in the absence of any commercial or financial relationships that could be construed as a potential conflict of interest.
